# General Transcription Factor IIF Polypeptide 2: A Novel Therapeutic Target for Depression Identified Using an Integrated Bioinformatic Analysis

**DOI:** 10.3389/fnagi.2022.918217

**Published:** 2022-05-27

**Authors:** Chi Zhang, Min Cheng, Naifu Dong, Dongjie Sun, Haichun Ma

**Affiliations:** ^1^Department of Anesthesiology, The First Hospital of Jilin University, Changchun, China; ^2^College of Basic Medical Sciences, Jilin University, Changchun, China

**Keywords:** depression, GTF2F2, therapeutic target, bioinformatics, WGCNA

## Abstract

Depression currently affects 4% of the world’s population; it is associated with disability in 11% of the global population. Moreover, there are limited resources to treat depression effectively. Therefore, we aimed to identify a promising novel therapeutic target for depression using bioinformatic analysis. The GSE54568, GSE54570, GSE87610, and GSE92538 gene expression data profiles were retrieved from the Gene Expression Omnibus (GEO) database. We prepared the four GEO profiles for differential analysis, protein–protein interaction (PPI) network construction, and weighted gene co-expression network analysis (WGCNA). Gene Ontology functional enrichment and Kyoto Encyclopedia of Genes and Genomes metabolic pathway analyses were conducted to determine the key functions of the corresponding genes. Additionally, we performed correlation analyses of the hub genes with transcription factors, immune genes, and N6-methyladenosine (m6A) genes to reveal the functional landscape of the core genes associated with depression. Compared with the control samples, the depression samples contained 110 differentially expressed genes (DEGs), which comprised 56 downregulated and 54 upregulated DEGs. Moreover, using the WGCNA and PPI clustering analysis, the blue module and cluster 1 were found to be significantly correlated with depression. *GTF2F2* was the only common gene identified using the differential analysis and WGCNA; thus, it was used as the hub gene. According to the enrichment analyses, GTF2F2 was predominantly involved in the cell cycle and JAK-STAT, PI3K-Akt, and p53 signaling pathways. Furthermore, differential and correlation analyses revealed that 9 transcription factors, 12 immune genes, and 2 m6A genes were associated with GTF2F2 in depression samples. GTF2F2 may serve as a promising diagnostic biomarker and treatment target of depression, and this study provides a novel perspective and valuable information to explore the molecular mechanism of depression.

## Introduction

Depression is a common mental health disorder that currently affects 4% of the world’s population, and it is associated with disability in 11% of the global population. It has been recognized as one of the most serious public health concerns with limited global public health progression (Hao et al., 2019). In some cases, psychiatric symptoms such as hallucinations and delusions may occur, accompanied by suicidal behavior (Chambers et al., 1982). The prevalence rate of depression in the United States of America exceeds 24% and is associated with other health and behavioral issues, including 800,000 annual suicides predominantly among young people. Millions of people die of the disease every year (Maurer et al., 2018). Moreover, depression increases the likelihood of early mortality, substance use, and anxiety (Casey, 2017; Kandola et al., 2019). Although the clinical symptoms of depression can be alleviated, the treatment and recurrence rates of the disease are not very optimistic because of patients’ inadequate knowledge and unwillingness to obtain regular treatments such as psychological and pharmacological therapies (Hardeveld et al., 2010). Indeed, half of the patients with major depressive disorder (MDD) do not respond to antidepressants (Hennings et al., 2009). To date, the specific cause of depression is not clear, and the dynamics and potential mechanisms of its onset and development remain poorly understood, thereby impeding the development and application of treatment strategies.

The recent rapid development of bioinformatics with high-throughput gene expression detection and hub gene screening methods, as well as weighted gene co-expression network analysis (WGCNA), has been advantageous in identifying the genes or specific molecular cascades involved in complex diseases, providing strategies for elucidating the molecular mechanisms of depression. Some molecular characterization and gene signatures have been shown to have pathophysiological significance in the mechanism of depression (Iwamoto et al., 2004; Bian et al., 2021; see also Liu et al., 2020). However, their biological functions need to be clarified, and integrated bioinformatic analyses of the transcription factors (TFs) and immune and methyladenosine gene characteristics in depression are still lacking.

In this study, four gene expression data profiles were retrieved from the GEO database, with an aim to discern some promising biomarkers in depression using integrative bioinformatic methods. We then conducted Gene Ontology (GO) functional enrichment and Kyoto Encyclopedia of Genes and Genomes (KEGG) metabolic pathway enrichment analyses of the core differentially expressed genes (DEGs) to estimate the underlying functions of the corresponding genes. After WGCNA and protein–protein interaction (PPI) analysis, general transcription factor IIF polypeptide 2 (*GTF2F2*) was identified as the hub gene. GTF2F2 combines with general transcription factor IIF polypeptide 1 (GTF2F1) to form a heteromeric general transcription initiation factor (TFIIF), which subsequently binds to DNA-dependent RNA polymerase II (Sasaki et al., 2013; Tsymbal et al., 2016). RNA polymerase II plays a critical role in mRNA synthesis, and GTF2F2 is essential for the initiation and elongation phases of gene transcription (Sasaki et al., 2013). Moreover, GTF2F2 is responsible for neurogenesis, neuroplasticity, and synaptogenesis by the mediation of Nuclear respiratory factor-1 (NRF-1) expression (Tong et al., 2013). Recently, GTF2F2 has also been reported to be involved in the potential development of SARS-CoV-2 (Pierzynowska et al., 2020). Therefore, *GTF2G2* was further analyzed, and the correlation of GTF2G2 with TFs, immune genes, and m6A genes was investigated.

## Materials and Methods

### Data Collection

The GSE54568, GSE54570, GSE87610, and GSE92538 gene expression data profiles were collected from the GEO database. These four profiles contained 15, 13, 72, and 56 normal and 15, 13, 76, and 29 depression samples, respectively. The R packages “limma” and “sva” were used to combine the four GEO profiles and to normalize the data matrix. Thereafter, the R package “ggplot2” was used to present sample distribution.

### Differential Analysis

The R packages “limma” and “impute” were utilized to identify the DEGs in the normal and depression samples. Furthermore, the cutoff criteria used were adjusted *p*-value < 0.05 and log_2_| fold change| > 0.5. The DEGs were then presented in a volcano plot and heat map.

### Weighted Gene Co-expression Network Analysis

We also conducted WGCNA using the R package “WGCNA” to select genes of interest. WGCNA transformed the adjacency matrix into a topological overlap matrix (TOM) in accordance with the soft threshold power. Subsequently, the genes were divided into different modules using the TOM-based dissimilarity measure. The cutoff threshold for minModuleSize and mergeCutHeight was set to 30 and 0.25, respectively.

### Protein–Protein Interaction Network Construction

The interaction information of proteins with a combined score > 0.7 was obtained from the search tool for the Retrieval of Interacting Genes/Proteins database.^1^ Cytoscape software (version 3.8.2) and its plugin, MCODE, were used to visualize the network and conduct clustering analysis of the network.

### Functional and Pathway Enrichment Analyses

FunRich software (version 3.1) was used to perform biological process and pathway enrichment analyses of the corresponding gene sets. Additionally, the R packages “org.Hs.eg.db” and “clusterProfiler” were used to conduct the GO and KEGG enrichment analyses of certain gene sets. Database for Annotation, Visualization, and Integrated Discovery (DAVID)^2^ was used to investigate the enrichment of genes in TFs.

### Correlation Analysis

The correlation information of genes was retrieved using the R package “corrplot” and the correlation heat map was plotted with the R package “heatmap.” The R packages “ggplot2,” “ggpubr,” and “ggExtra” were used to plot correlation density images. Moreover, the depression samples were grouped into high and low hub gene subgroups based on the median expression of the hub genes. We also used the R packages “limma” and “vioplot” to visualize the violin plots for the DEGs.

## Results

### General Transcription Factor IIF Polypeptide 2 Identified as the Hub Gene

**FIGURE 1:**
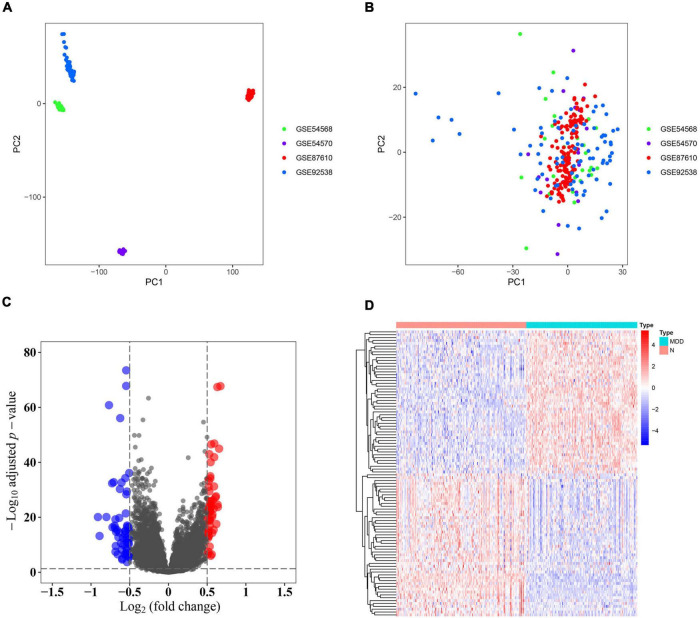
Identification of differentially expressed genes (DEGs). **(A)** Principal component analysis (PCA) of the four profiles (GSE54568, GSE54570, GSE87610, and GSE92538) was conducted using the pre-normalized data matrix. **(B)** PCA of the four profiles (GSE54568, GSE54570, GSE87610, and GSE92538) was performed using the normalized data matrix. **(C)** Volcano plot of the DEGs in normal and depression samples, wherein the blue and red dots represent downregulated and upregulated DEGs, respectively. **(D)** Heat map plot of the expression profile of the DEGs in normal and depression samples.

Samples from the four GEO profiles (GSE54568, GSE54570, GSE87610, and GSE92538) were uniformly and logically scattered in the principal component analysis (PCA) plot after normalization using the R package (as shown in Figures 1A,B). Compared with the normal samples, the depression samples contained 110 DEGs, which comprised 56 downregulated and 54 upregulated DEGs (as shown in Figure 1C). These DEGs presented different expression profiles in patients with depression compared with those in the corresponding normal controls (as shown in Figure 1D). The DEGs were substantially enriched in energy pathways, protein metabolism, metabolism, and other biological processes (as shown in Supplementary Figure 1A and Supplementary Table 1). For the biological pathway, the DEGs were predominantly associated with the adaptive immune system, interferon signaling, and antigen processing-cross presentation (as shown in Supplementary Figure 1B and Supplementary Table 2).

**FIGURE 2:**
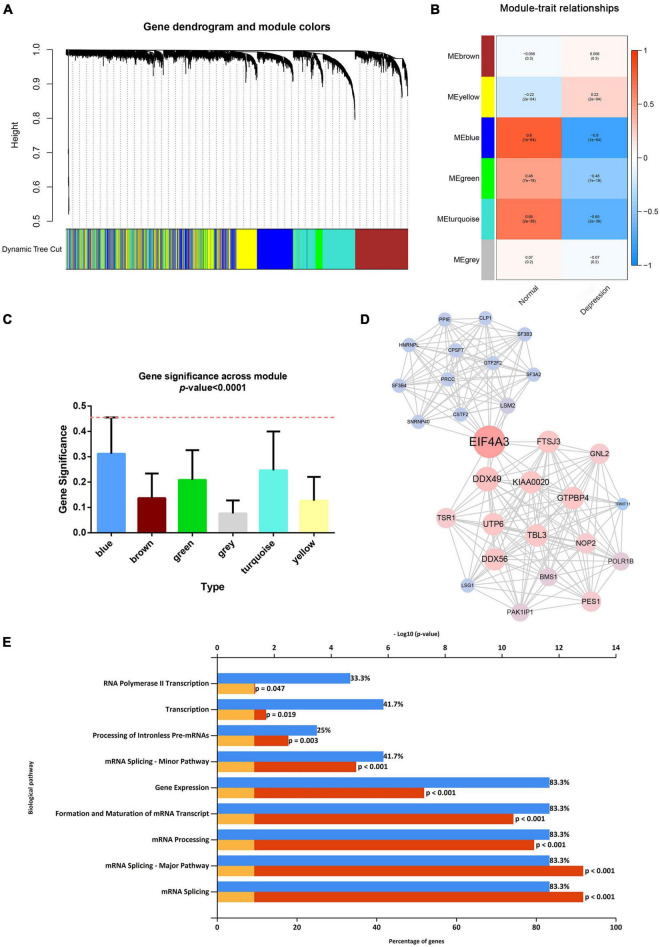
Weighted gene co-expression network analysis and protein–protein analysis. **(A)** Image of a clustering dendrogram for the Gene Expression Omnibus profiles using the dissimilarity measure 1-TOM. **(B)** Heat map of the correlation between the module eigengenes and sample type. **(C)** Histogram for module significance of the distribution of the average gene significance and module errors for patients with depression. **(D)** Image of the network of the highest-scoring cluster in the protein–protein interaction network. **(E)** The typical pathways are enriched by the differentially expressed genes in the highest-scoring cluster.

After considering the distribution of clinical traits within all 289 samples, no outliers were removed when the samples were clustered according to the WGCNA and differential analysis (as shown in Supplementary Figure 1). A soft-thresholding power of 10 and a scale-free *R*^2^ of 0.80 were identified and used to construct a scale-free network (as shown in Supplementary Figures 3A,B). Subsequently, six modules were identified; the blue module was significantly correlated with depression, and it presented a higher module significance than other modules (as shown in Figures 2A–C). Genes within the blue module were used to construct a PPI network containing 284 nodes and 764 edges (as shown in Supplementary Figure 4). We then analyzed the three clusters with the highest clustering scores within the PPI network, of which cluster 1 with the highest clustering score (14.427) comprised 29 nodes and 202 edges (as shown in Figure 2D) compared to cluster 2 (as shown in Supplementary Figure 5), and cluster 2 (as shown in Supplementary Figure 6). Cluster 1 was associated with mRNA splicing, processing, and formation; mRNA transcript maturation; and other pathways (as shown in Figure 2E). Additional details are provided in Supplementary Tables 3–5.

**FIGURE 3:**
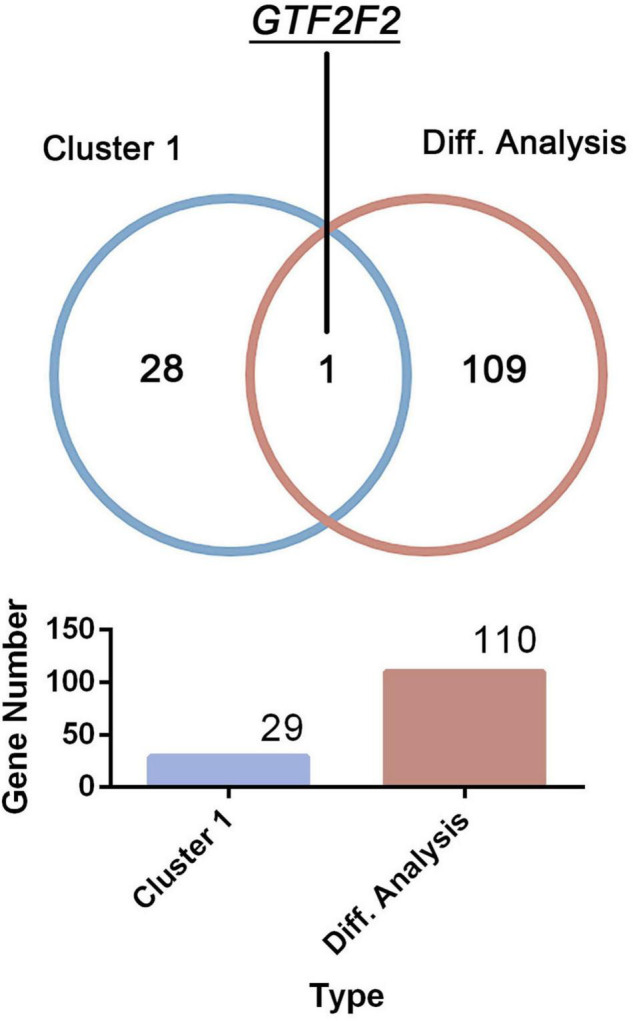
Determination of the core gene. *GTF2F2* was the only common gene identified using the clustering analysis within cluster 1 and the differential analysis of both normal and depression samples.

Only one common gene, *GTF2F2*, was identified using the DEGs in the differential analysis and genes within cluster 1 (as shown in Figure 3). Therefore, *GTF2F2* was used as the hub gene in this study.

### Functional Analysis of General Transcription Factor IIF Polypeptide 2

**FIGURE 4:**
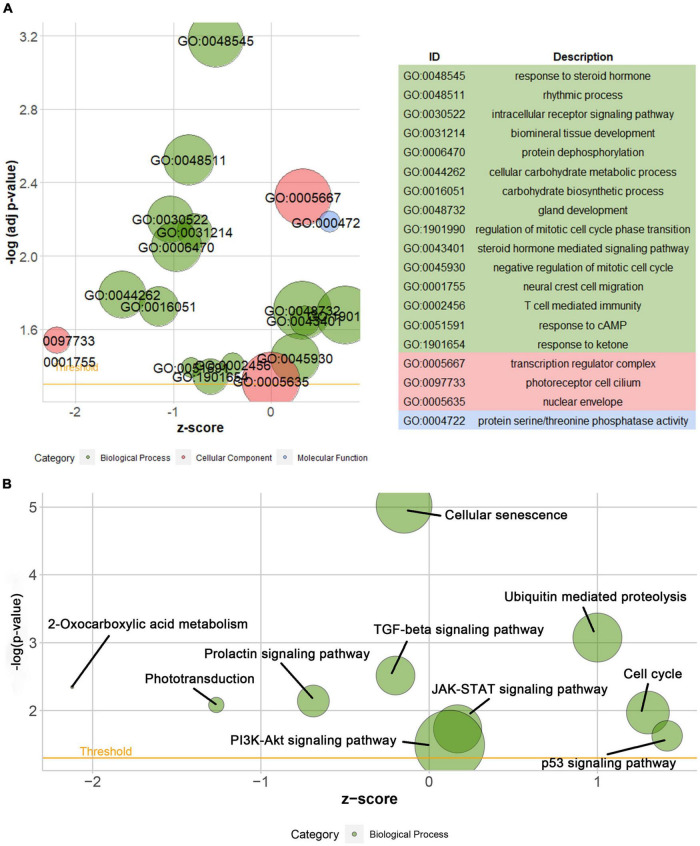
Gene Ontology (GO) and Kyoto Encyclopedia of Genes and Genomes (KEGG) enrichment analyses of GTF2F2 function. **(A)** Bubble plot of the GO function analysis of the differentially expressed genes (DEGs) in the low and high GTF2F2 subgroups (grouped according to the median GTF2F2 expression levels). **(B)** Bubble plot of the KEGG pathway analysis of the DEGs in the low and high GTF2F2 expression subgroups. A higher z-score represents a higher expression of enriched terms.

**FIGURE 5:**
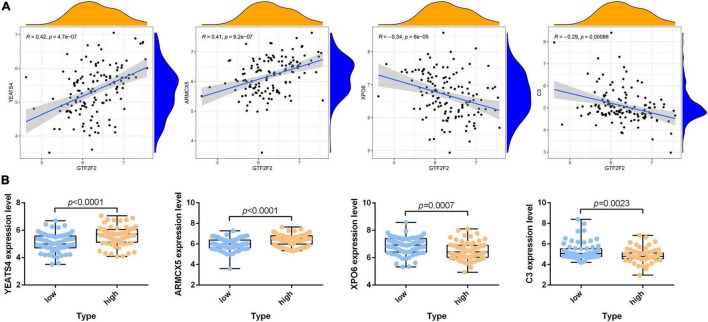
Correlation of *GTF2F2* with *YEATS4*, *ARMCX5*, *XPO6*, and *C3*. **(A)** Scatter plots of the correlation of *GTF2F2* with *YEATS4*, *ARMCX5*, *XPO6*, and *C3*. **(B)** Histograms of the expression profiles of *YEATS4*, *ARMCX5*, *XPO6*, and *C3* in the low and high GTF2F2 subgroups (grouped according to the median GTF2F2 expression levels).

According to the differential analysis results, *GTF2F2* was significantly upregulated in depression samples compared with that in the corresponding controls (as shown in Supplementary Figure 7A), and the depression samples were grouped into high and low GTF2F2 expression subgroups based on the median GTF2F2 expression levels (as shown in Supplementary Figure 7B). Additionally, the DEGs in the high and low GTF2F2 expression subgroups were enriched using R software to determine the potential functions of GTF2F2. We found that GTF2F2 was significantly associated with responses to steroid hormones, rhythmic processes, intracellular receptor signaling pathways, and other biological processes (as shown in Figure 4A). The transcription regulator complex, photoreceptor cell cilium, and nuclear envelope were the main cellular components enriched. In terms of molecular function, GTF2F2 was only related to protein serine/threonine phosphatase activity. Further details are provided in Supplementary Table 6. Furthermore, GTF2F2 may be involved in the cell cycle and cell senescence. It may also be involved in JAK-STAT, PI3K-Akt, p53, TGF-β, and other biological signaling pathways (as shown in Figure 4B). Additional details are provided in Supplementary Table 7. Moreover, a heat map was plotted to depict the association between the top 10 downregulated and upregulated DEGs in the high GTF2F2 subgroup, compared with that in the low GTF2F2 subgroup (as shown in Supplementary Figure 8). *YEATS4* and *ARMCX5* had a positive correlation with *GTF2F2*, whereas *XPO6* and *C3* had a negative correlation with *GTF2F2* (as shown in Figure 5).

### General Transcription Factor IIF Polypeptide 2 Associated With Transcription Factors

**FIGURE 6:**
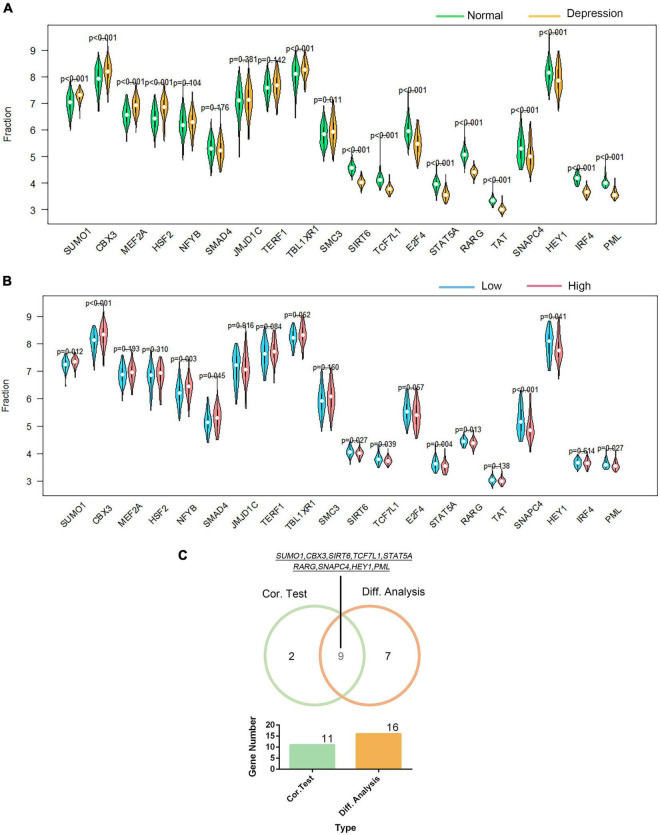
Correlation of GTF2F2 with transcription factors. **(A)** Violin plot of the differential analysis of the top 20 transcription factors (TFs) in non-psychiatric control samples and depression samples. The top 20 TFs were determined using the correlation coefficient of GTF2F2 with TFs. **(B)** Violin plot of the expression profiles of the top 20 TFs in the subgroups with low and high GTF2F2 expression levels. **(C)** Venn plot of the nine common TFs that overlapped in the normal and depression samples and the subgroups with low and high GTF2F2 expression levels.

Using the DAVID platform, the 20 DEGs in the correlation analysis were found to be predominantly enriched in the five TFs: RORA2, TCF11, PPARA, GATA2, and LMO2COM (as shown in Supplementary Figure 9). Additionally, we established the correlation between GTF2F2 and TFs based on their mRNA expression levels, whereby the TFs with the top 10 positive and negative correlations are depicted in a heat map (as shown in Supplementary Figure 10). We used 20 TFs for further analysis, 16 of which were significantly differentially expressed in depression samples compared with those in normal samples as shown in Figure 6A, and 11 TFs were significantly related to GTF2F2, based on their expression levels in the high and low GTF2F2 expression subgroups (as shown in Figure 6B). Moreover, nine TFs overlapped in the differential and correlation analyses (as shown in Figure 6C).

### General Transcription Factor IIF Polypeptide 2 Associated With Immune Genes

**FIGURE 7:**
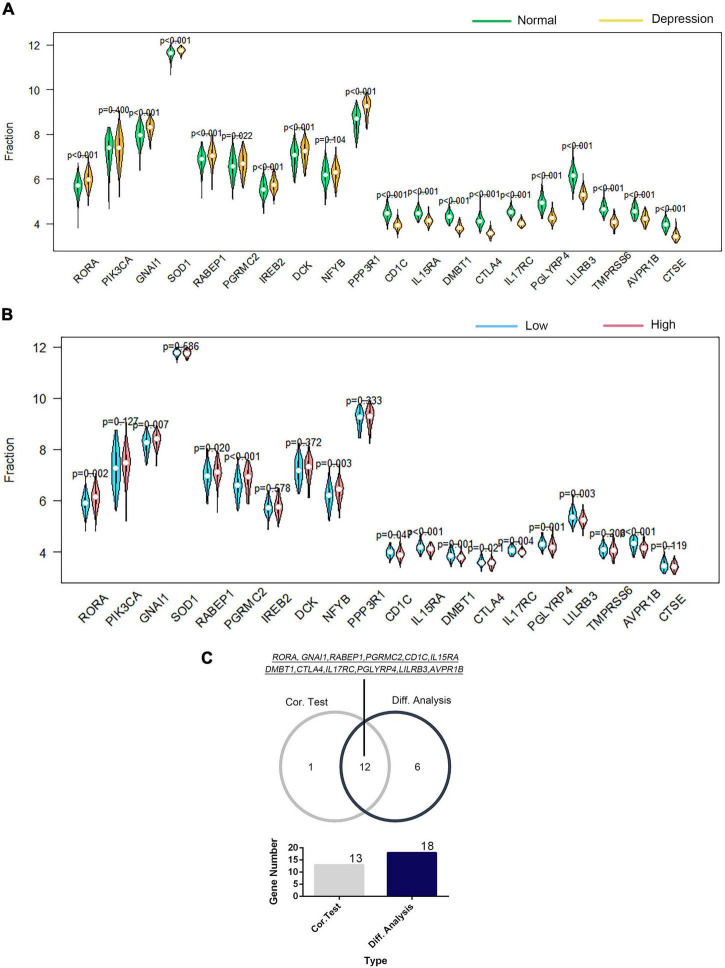
Correlation of GTF2F2 with immune genes. **(A)** Violin plot of the differential analysis of the top 20 immune genes in normal and depression samples. The top 20 immune genes were determined using the correlation coefficient of GTF2F1 with immune genes. **(B)** Violin plot of the expression profiles of the top 20 immune genes in the subgroups with low and high GTF2F2 expression levels. **(C)** Venn plot of the 12 common immune genes that overlapped between the normal and depression samples and between the subgroups with low and high GTF2F2 expression levels.

The correlation of GTF2F2 with immune genes was also determined based on their mRNA expression levels using R software, and the genes with the top 10 positive and negative association coefficients are presented in Supplementary Figure 11. Twenty immune genes were utilized to perform further differential and correlation analyses; 18 immune genes were significantly differentially expressed in depression samples compared with those in the corresponding controls (as shown in Figure 7A), and 13 immune genes were significantly associated with GTF2F2 based on their expression levels in the high and low GTF2F2 expression subgroups as shown in Figure 7B. Furthermore, 12 genes overlapped in the differential and correlation analyses (as shown in Figure 7C).

### General Transcription Factor IIF Polypeptide 2 Associated With N6-Methyladenosine Genes

**FIGURE 8:**
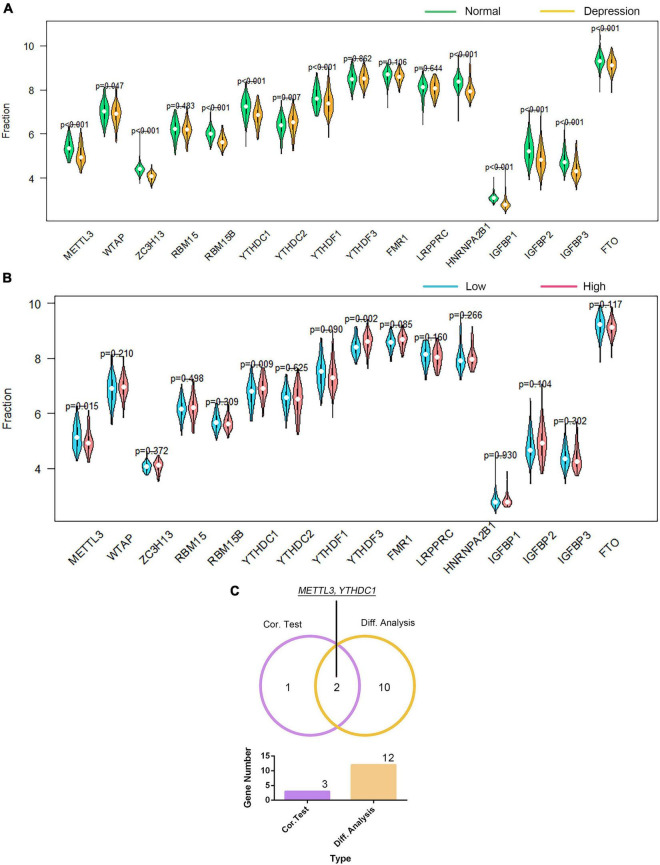
Correlation of GTF2F2 with *m6A* genes. **(A)** Violin plot of the differential analysis of the top 20 *m6A* genes in normal and depression samples. The top 20 *m6A* genes were determined using the correlation coefficient of GTF2F2 with *m6A* genes. **(B)** Violin plot of the expression profiles of the top 20 *m6A* genes in the subgroups with low and high GTF2F2 expression levels. **(C)** Venn plot of the two common *m6A* genes that overlapped between the normal and depression samples and between the subgroups with low and high GTF2F2 expression levels.

We investigated the association of GTF2F2 with m6A genes; Supplementary Figure 12 presents a heat map of the correlation between 20 m6A genes with the top 10 positive and negative correlation coefficients. The differential and correlation analyses revealed that 12 m6A genes were significantly different in depression samples compared with those in their corresponding controls (as shown in Figure 8A). Moreover, three m6A genes were significantly correlated with GTF2F2 based on their expression levels in the high and low GTF2F2 expression subgroups (as shown in Figure 8B). Additionally, *METTL3* and *YTHDC1* were the only overlapping genes identified in the differential and correlation analyses (as shown in Figure 8C).

## Discussion

Depression with heterogeneous characteristics has been poorly reported, diagnosed, and treated. Patients with depression are suspected of being socially abandoned and having reduced interactions with others to manage their complex matters. Depression also affects the educational status, professional lives, and romantic relationships of individuals during their transition to adulthood (Park et al., 2018). In particular, MDD becomes a serious mental disorder that profoundly affects an individual’s quality of life. Most patients with MDD are not effectively treated, and it is not clear why they discontinue treatment. The low response rates and high adverse effect burden of antidepressants mean that depression would require a longer time to attain therapeutic benefits. To date, there is no efficient biomarker for early diagnosis or for predicting or monitoring the pathogenesis of MDD in clinical trials, because the etiology and pathogenic mechanisms are undefined. Patients with depression have been reported to have reduced gray matter volume in the dorsolateral prefrontal cortex (DLPFC), and this may be associated with suicidal ideation (Zhang et al., 2020). Moreover, DLPFC-mediated cognitive control may also be related to emotional regulation, cognitive dysfunction, and motivation (Jones and Graff-Radford, 2021). Therefore, we collected the datasets containing DLPFC tissues of MDD, with an aim to identify a promising novel therapeutic target using an integrated bioinformatic analysis to provide valuable findings for further examinations of the pathogenic mechanisms of depression.

In our study, GTF2F2 was identified as the hub gene associated with MDD. GTF2F2 has a potential role in regulating neurite outgrowth. Neurite outgrowth is an intricate process, regulated by numerous genes. It is reported that GTF2F2 positively regulates neurite outgrowth through NRF-1 mediation. After silencing NRF-1, the mRNA levels of GTF2F2 increased, whereas the knockdown of GTF2F2 significantly decreased NRF-1-regulated neurite outgrowth (Tong et al., 2013; Preciados et al., 2016). Our results showed that GTF2F2 was significantly associated with rhythmic processes. Dysregulation of the circadian system increases the susceptibility to pathological conditions, including depression. Over 90% of the patients have sleep complaints. The successful treatment of depression frequently leads to an improvement in altered circadian rhythm (Satyanarayanan et al., 2018; Geoffroy and Palagini, 2021). Furthermore, our results illustrated that higher expression of GTF2F2 foreboded higher expression of retinoid-related orphan receptor alpha (RORA), which has been linked to MDD (Ming et al., 2015; Min et al., 2017). RORA is involved in the regulation of circadian rhythms, and it has been suggested to be correlated with depression vulnerability (Chen et al., 2021). GTF2F2 may affect pathogenesis of depression through the circadian process. However, further verification needs to be carried out for identification.

Depression is associated with abnormalities in the immune system. The central nervous system, endocrine system, and immune system share the same neurotransmitters, cytokines, and hormones to communicate within and among each other (Kovaru et al., 2009). There is considerable evidence that inflammatory response and immune system changes are a part of depression. Inflammation is likely a critical disease modifier, promoting susceptibility to depression (Leonard, 2001). We showed that GTF2F2 is involved in JAK-STAT, PI3K-Akt, p53, TGF-β, and other biological signaling pathways related to immune response. The transforming growth factor (TGF)-β pathway, with its role as a regulatory cytokine, has been involved in the pathophysiology of MDD (Lee and Kim, 2010). It is known that NRF-1, as an important regulator of GTF2F2, can interfere with the TGF-β/SMAD pathway with a novel negative regulation of SMAD4 (Rajasekaran et al., 2021). From our results, GTF2F2 may affect the TGF-β pathway through NRF-1, and therefore, associate with MDD progression. Besides, JAK-STAT signaling pathway was reported in the MDD (Gao et al., 2022; Maes et al., 2022), which may also act as a target of GTF2F2 affecting MDD.

Moreover, higher GTF2F2 levels were associated with lower IL15Rα receptor levels. Interleukin-15 affects the serotonin system and exerts anti-depressive effects through the IL15Rα receptor. Additionally, IL15Rα knockout mice show depressive phenotypes (Wu et al., 2011). RORA is also involved in the regulation of types 2 and 3 innate lymphoid cells, macrophages, and Treg cells (De Matteis et al., 2018; Lo et al., 2019; L’homme et al., 2020; Haim-Vilmovsky et al., 2021). Using overexpression in CD4-conditional *RORA*-knockout mice, Haim-Vilmovsky et al. (2021) investigated the role of RORA in regulating inflammation in CD4 + T cells and found that RORA could negatively regulate the immune system. Moreover, the RORA activity is regulated by interleukin-33, chemokine (CC motif) ligand 7, and the local microenvironment (Jiang et al., 2019; Yang et al., 2019; Haim-Vilmovsky et al., 2021; Zou et al., 2021). C3, the key molecule in the complement cascade reaction, is primarily produced by astrocytes (Lian et al., 2016). In an MDD model, CUMS mice, injected with a C3aR antagonist, accumulate the expression of C3a and C3aR, and the microglial polarization was observed (Li et al., 2020). Our result shows that high GTF2F2 has a negative correlation with C3, which may involve in the regulation process. These results revealed that GTF2F2 may play a role in the immune function of depressive behaviors, and suppression of GTF2F2 may be a way to effectively ameliorate depressive symptoms.

GTF2F2 is essential for the initiation and elongation phases of gene transcription (Sasaki et al., 2013; Tsymbal et al., 2016; Ramayo-Caldas et al., 2019). Sasaki et al. (2013) suggested that mutations in the 3′ UTR of GTF2F2 could influence the RNA expression of GTF2F2. Additionally, the allelic imbalance contributes to GTF2F2 expression, which may be responsible for the slight increase in RNA polymerase II activity (Sasaki et al., 2013). Moreover, GTF2F2, COPS4, PSMA6, GTF2B, and SSB have been identified as dysregulated TFs that are correlated with Alzheimer’s disease and diabetes mellitus. These five TFs are differentially expressed in the corresponding tissue, such as the brain in Alzheimer’s disease and the pancreas in diabetes mellitus (Lee and Lee, 2021). Lee and Lee found that *GTF2F2*, *COPS4*, *PSMA6*, *GTF2B*, and *SSB* comprised a gene module, which was dysregulated in blood samples of patients with Alzheimer’s disease and diabetes mellitus (Lee and Lee, 2021). GTF2F2 interacts with GTF2F1 to construct the TFIIF complex that is necessary to form RNA polymerase II, which is essential for the initiation and elongation of transcription (Aso et al., 1993; Purrello et al., 1995; Tsymbal et al., 2016). During transcription elongation, GTF2F2 competes with Gdown1, the substoichiometric 13th subunit of RNA polymerase II, and it is involved in pausing the early stage of elongation to regulate the activity of RNA polymerase II.

TFs, a group of DNA-binding proteins, are essential for the molecular state of a cell (Goossens et al., 2017; Li and Leonard, 2018; Tian et al., 2018; Vishnoi et al., 2020). Several studies have recognized the importance of understanding the behaviors and functions of TFs (Füllgrabe and Moore, 2018; Martínez Corrales and Alic, 2020). For example, HEY1, a transcription factor, is an effector molecule of the NOTCH signaling pathway (Watanabe et al., 2020; Xie et al., 2020). In salivary adenoid cystic carcinoma, NOTCH is positively associated with HEY1 activation, and HEY1 affects the expression of NOTCH and contributes to cellular proliferation, apoptosis inhibition, and spheroid formation *in vitro* (Xie et al., 2020). Additionally, recent studies have revealed that the immune system plays pivotal roles in brain function, such as learning and memory, and in positively regulating neural plasticity and neurogenesis (Dougherty et al., 1987; Dantzer, 2018; Besedovsky, 2019). Recently, several studies have demonstrated the essential functions of post-translational modifications, particularly in influencing the transcriptomic landscape of the brain (Hendee et al., 2017; Park et al., 2018; Elsamadicy et al., 2021). Additionally, methylation of the adenosine base at the N6 position is the most common internal modification shaping eukaryotic mRNAs (Widagdo and Anggono, 2018); m6A exerts additional regulatory effects on RNA in a context- and stimulus-dependent manner (Chen et al., 2019; Zhao et al., 2020). GTF2F2 is correlated with post-transcriptional modification in protein molecules, including *METTL3* and *YTHDC1*. The abnormal expression of m6A-related proteins occurs in the nervous system, thereby affecting neuritogenesis, brain volume, learning and memory, memory formation and consolidation, and is implicated in the pathogenesis of depression (Zhang et al., 2022). This revealed that GTF2F2 might act as a satisfactory indicator for abundant mRNA modifications that regulate transcript processing and translation in depression.

This study contributes to our understanding of the potential mechanisms underlying depression and provides novel insights into the therapeutic strategies for this mental health condition. However, this study had some limitations. First, this study was solely conducted using bioinformatic analysis; thus, the results were not validated with new experimental data. Second, the molecular mechanisms of GTF2F2 in depression and the correlation of GTF2F2 with the corresponding genes, such as the key functions of GTF2F2 in its interactions with the immune system and m6A genes, were not examined in detail. Finally, GTF2F2 was identified as a core gene for depression using GEO profiles with small sample sizes and imbalanced clinical data. Therefore, these limitations should be addressed in future research.

## Conclusion

Using an integrated bioinformatical analysis, for the first time to our knowledge, *GTF2F2* was identified as a hub gene that may be vital for the onset and development of depression, which may serve as a promising novel indicator for the pathogenesis of depression. Further studies are needed to explore the correlation of GTF2F2 with immune response pathways such as the JAK-STAT and PI3K-Akt pathways and with inflammatory factors, which may improve the treatment of patients with depression.

## Data Availability Statement

The original contributions presented in the study are included in the article/Supplementary Material, further inquiries can be directed to the corresponding author/s.

## Author Contributions

HM and DS conceived and designed the experiments. CZ and MC conducted the acquisition, analysis, and interpretation of data. CZ and ND confirmed the authenticity of the raw data. CZ wrote the manuscript. All authors read and approved the final manuscript.

## Conflict of Interest

The authors declare that the research was conducted in the absence of any commercial or financial relationships that could be construed as a potential conflict of interest.

## Publisher’s Note

All claims expressed in this article are solely those of the authors and do not necessarily represent those of their affiliated organizations, or those of the publisher, the editors and the reviewers. Any product that may be evaluated in this article, or claim that may be made by its manufacturer, is not guaranteed or endorsed by the publisher.

## Footnotes

1
https://string-db.org/

2
https://david.ncifcrf.gov
