# Supplementary material for: General Transcription Factor IIF Polypeptide 2: A Novel Therapeutic Target for Depression Identified Using an Integrated Bioinformatic Analysis
Source: Front Aging Neurosci. 2022 May 27;14:918217. doi: 10.3389/fnagi.2022.918217 (PMC9197343; doi:10.3389/fnagi.2022.918217)
Supplement: Supplementary file 1 [file Data_Sheet_1.docx]

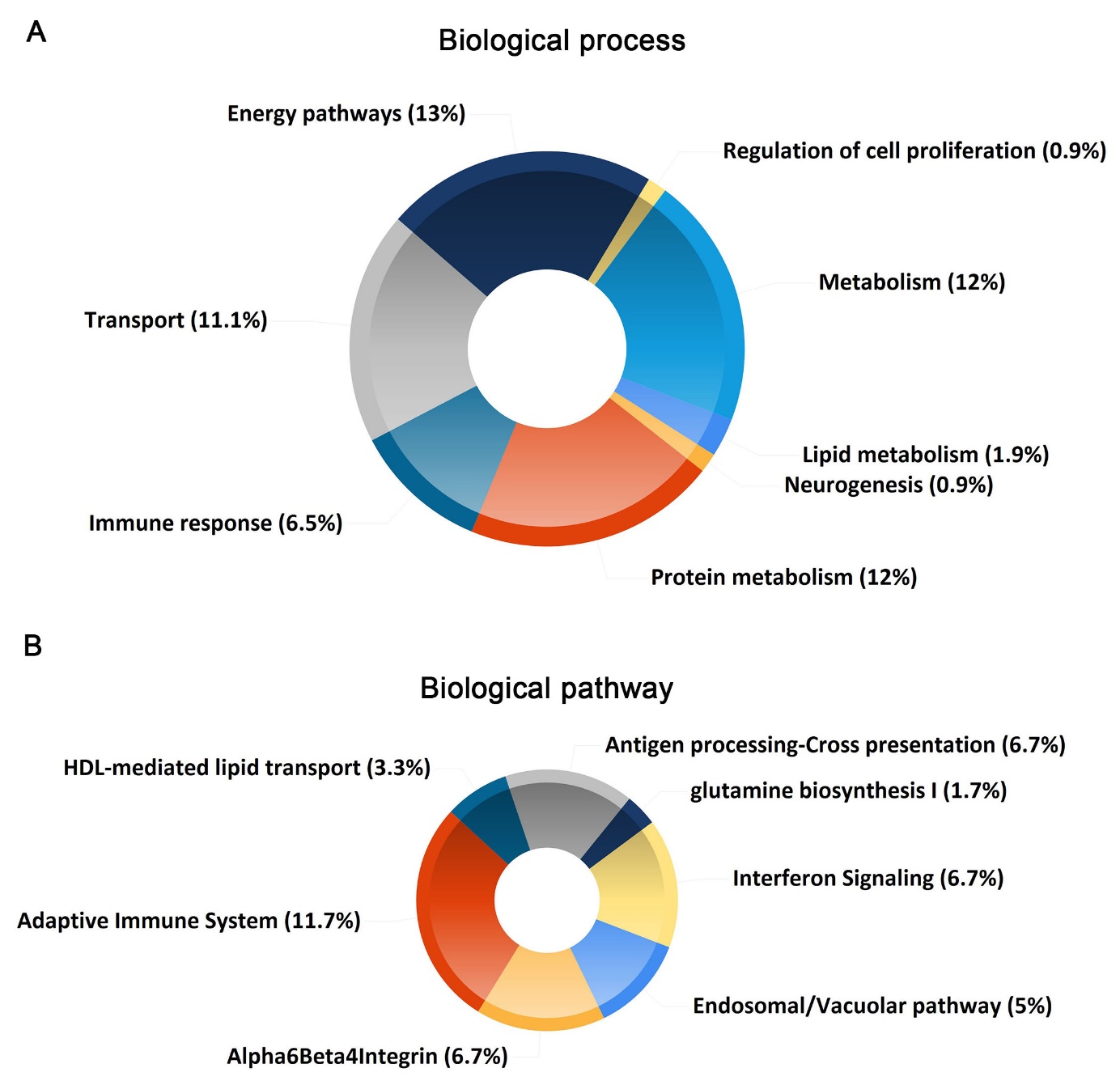


Figure S1. Enrichment analyses for the differentially expressed genes. Biological process enriched by the differentially expressed genes (DEGs) between normal and depression samples using software of FunRich (A). Pie plot implying the biological pathway were associated with the DEGs (B).


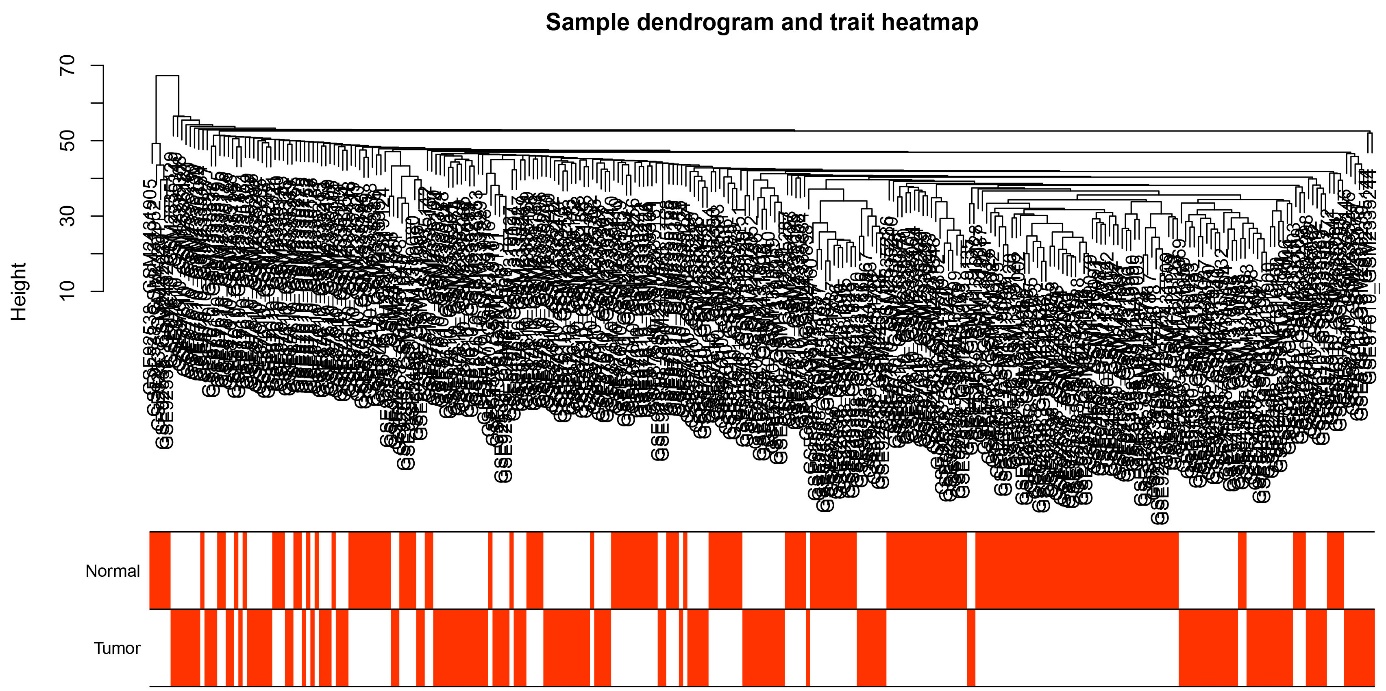


Figure S2. Sample clustering dendrogram and clinical traits indicator based on the expression data from the four GEO profiles (GSE54568, GSE54570, GSE87610 and GSE92538). The red color block corresponds to the clinical information of samples in the clustering diagram.


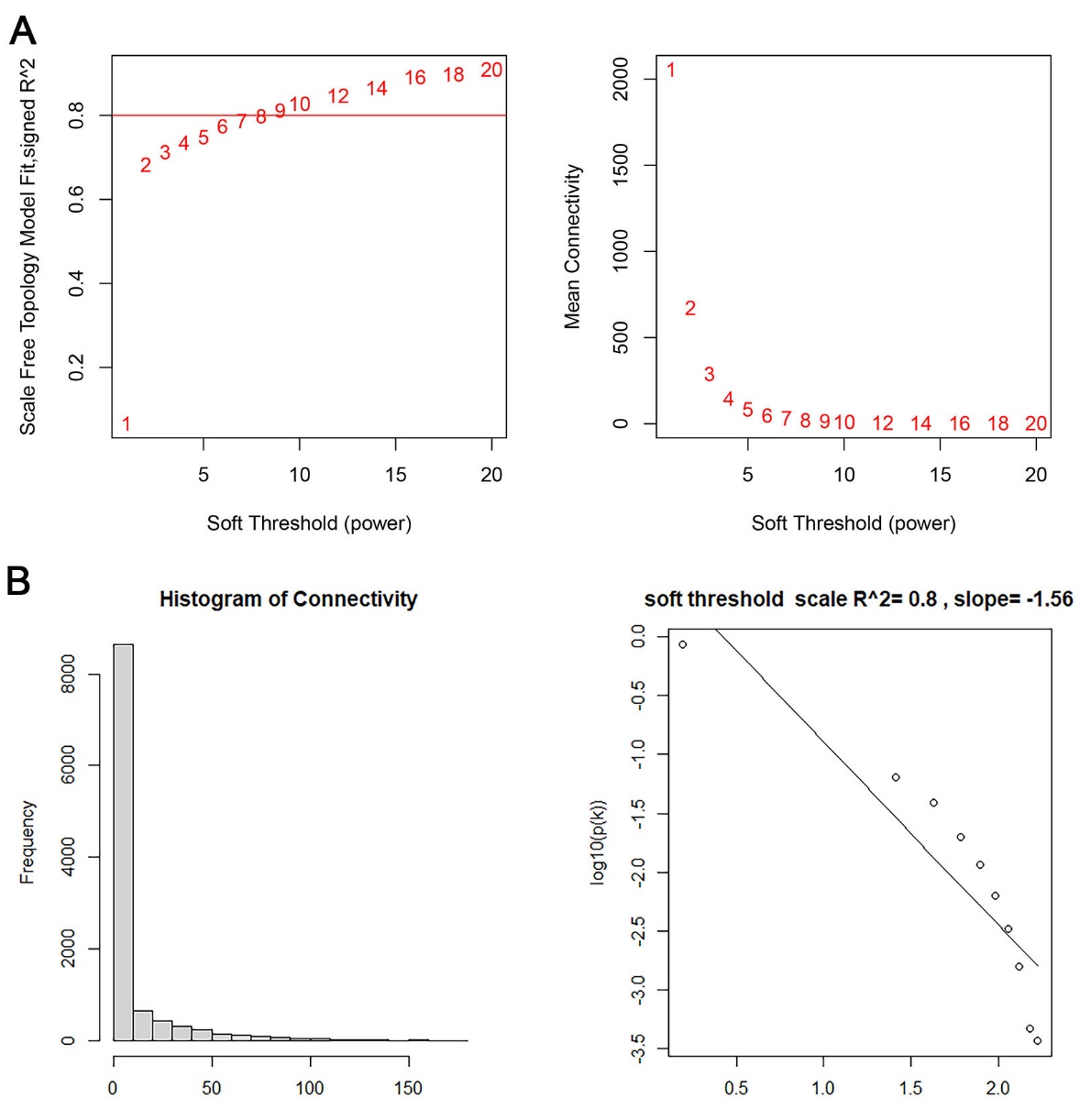


Figure S3. Determination of soft-thresholding power in the weighted gene co-expression network analysis (WGCNA). Topology analysis of the scale-free fit index (A) and the mean connectivity (B) for different soft-thresholding powers (β). Histogram of connectivity distribution (C) and scatter plot for checking the scale free topology (D) when β = 8.


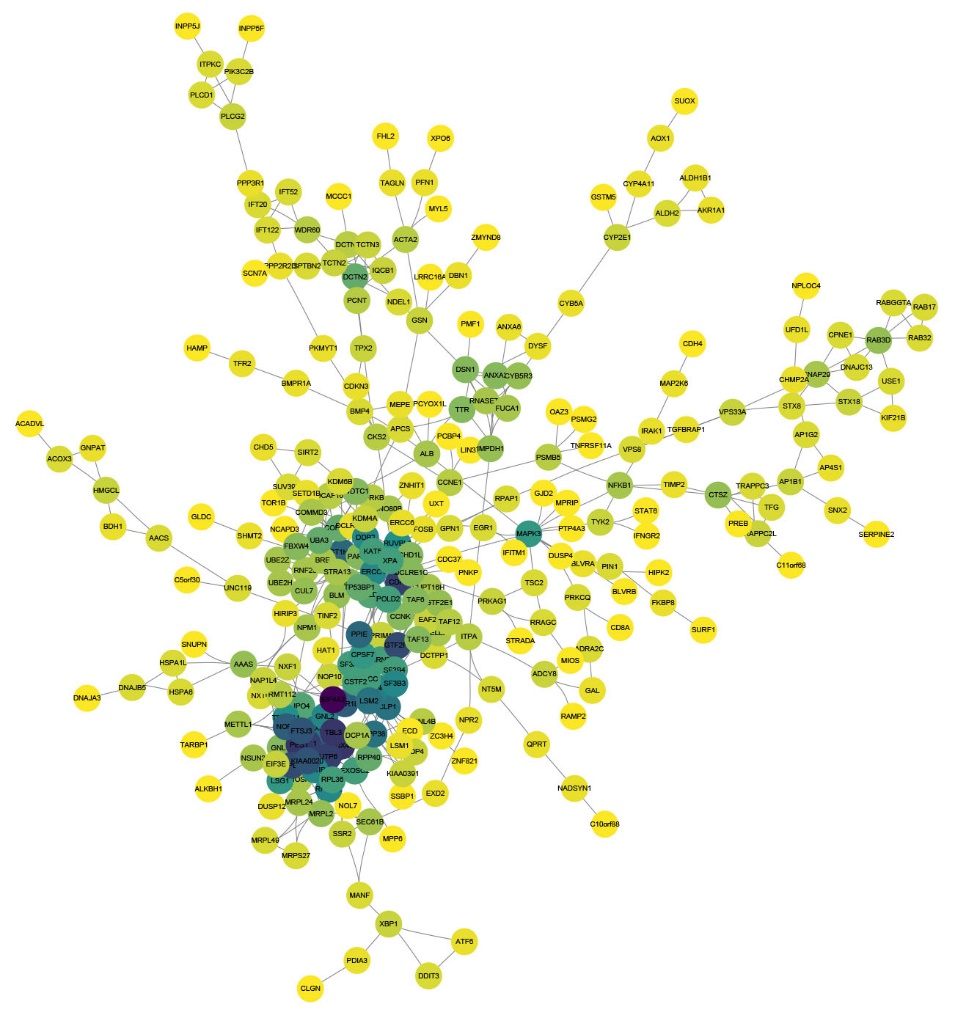


Figure S4. Protein-protein interaction (PPI) network. PPI network was conducted by genes in the blue model of WGCNA, which included 284 nodes and 764 edges.


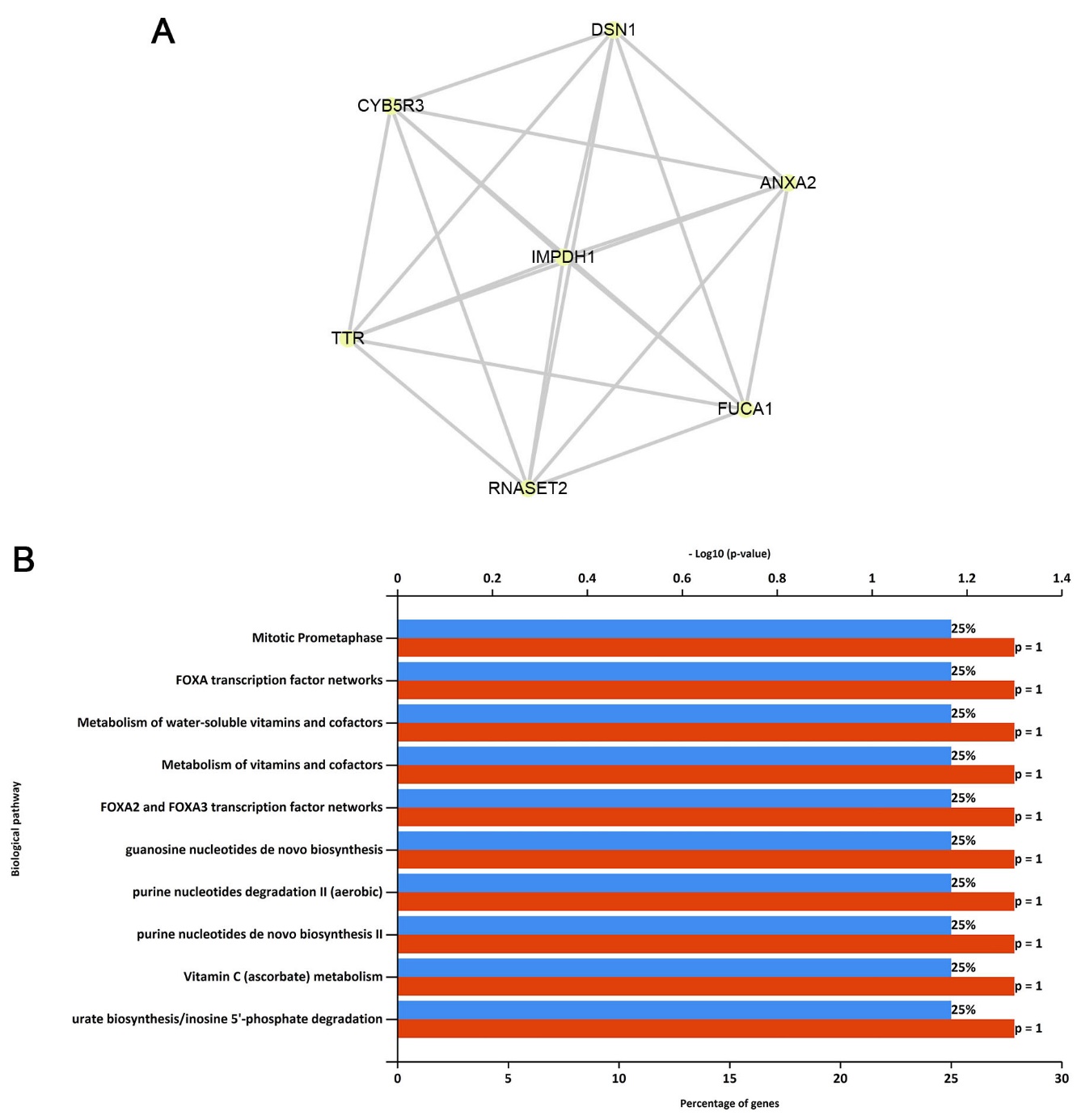


Figure S5. The typical pathways of cluster 2 in PPI network. Cluster 2 in PPI network was determined by the MCODE, a plug-in of cytoscape, which contained 7 nodes and 21 edges (A). The enrichment pathways by genes in cluster 2 was analyzed by the software FunRich (B).


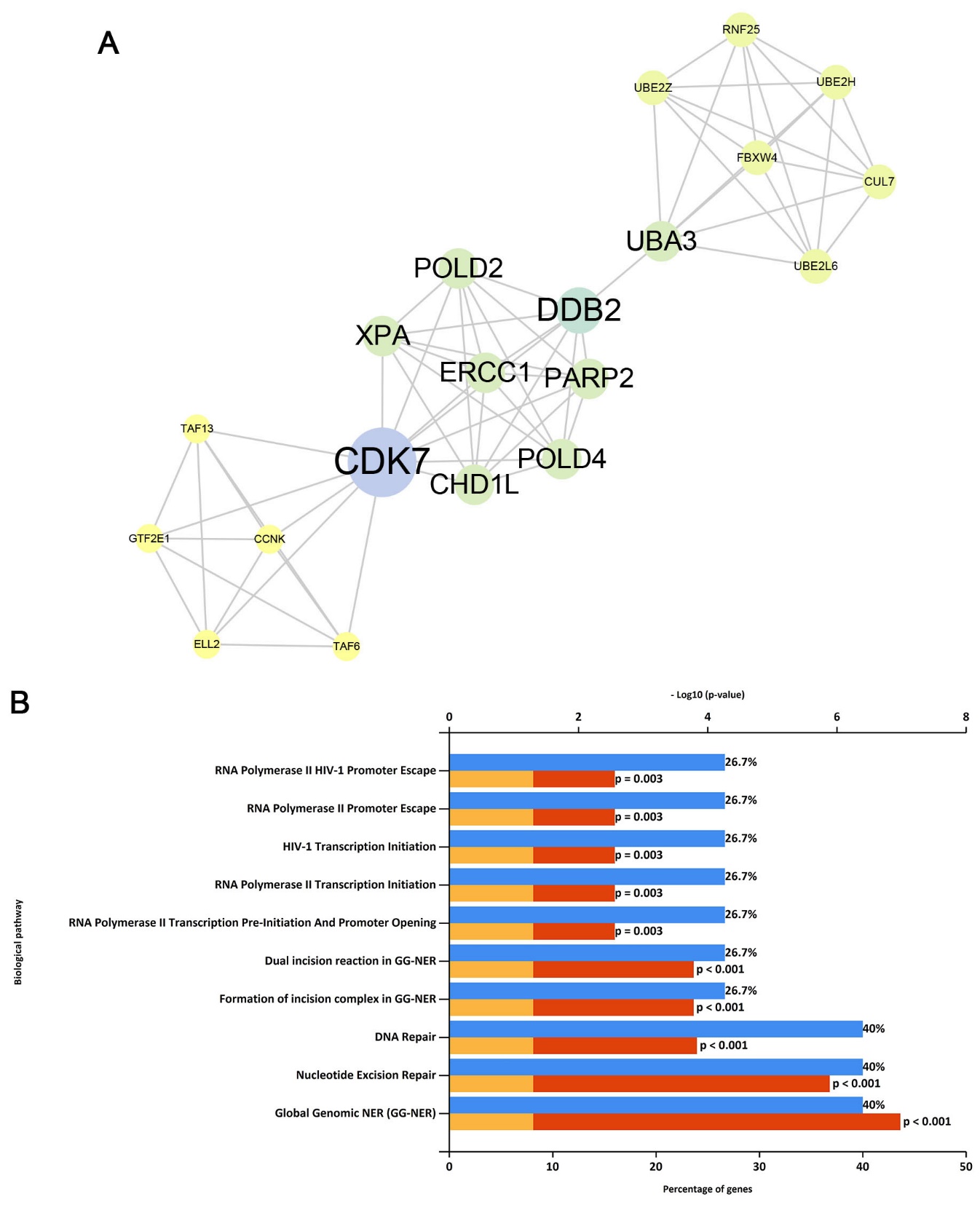


Figure S6. The typical pathways of cluster 3 in PPI network. Cluster 3 in PPI network was determined by the MCODE, which contained 20 nodes and 65 edges (A). The enrichment pathways by cluster 3 was performed by FunRich (B).


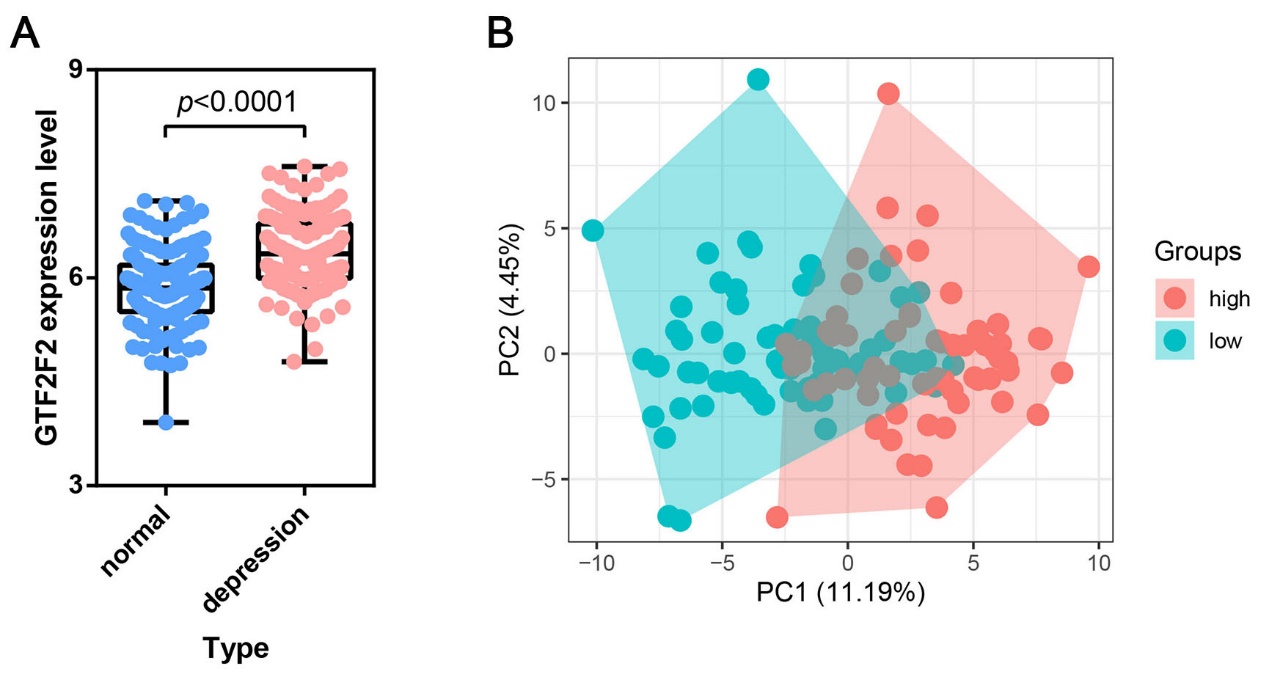


Figure S7. Identification of subgroups with low and high GTF2F2 expression. Scatter plot indicated the GTF2F2 expression with significantly statistical difference between normal and depression samples (A). PCA implied samples was classed into two subtypes with low and high GTF2F2 expression based on the median expression (B).


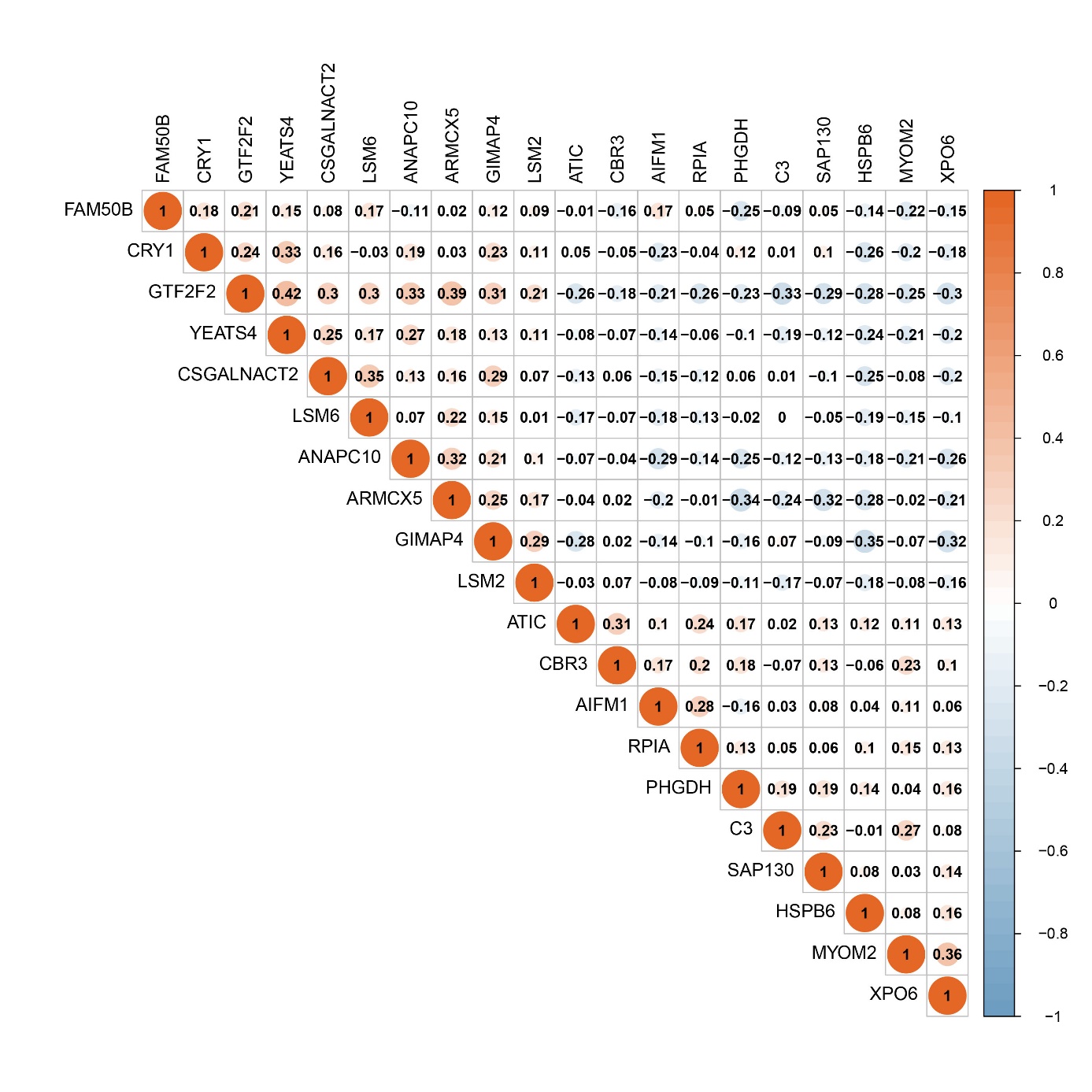


Figure S8. Correlation of GTF2F2 with differentially expressed genes (DEGs). Heatmap implied the association among top10 up-regulated and top10 down-regulated DEGs based on log2|fold change| in high GTF2F2 expression subgroup compared to low GTF2F2 expression subgroup.


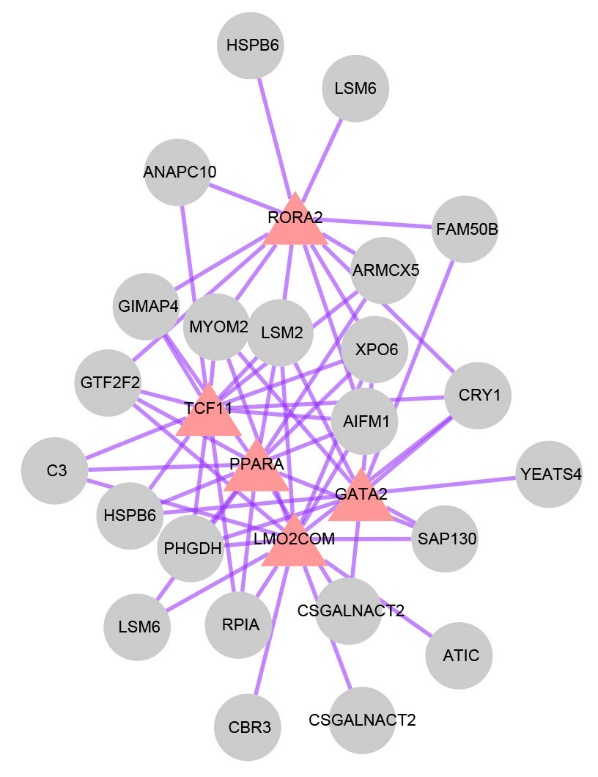


Figure S9. Association of DEGs with transcription factors (TFs). Network indicated the twenty DEGs had correlation with four TFs based on DAVID platform.


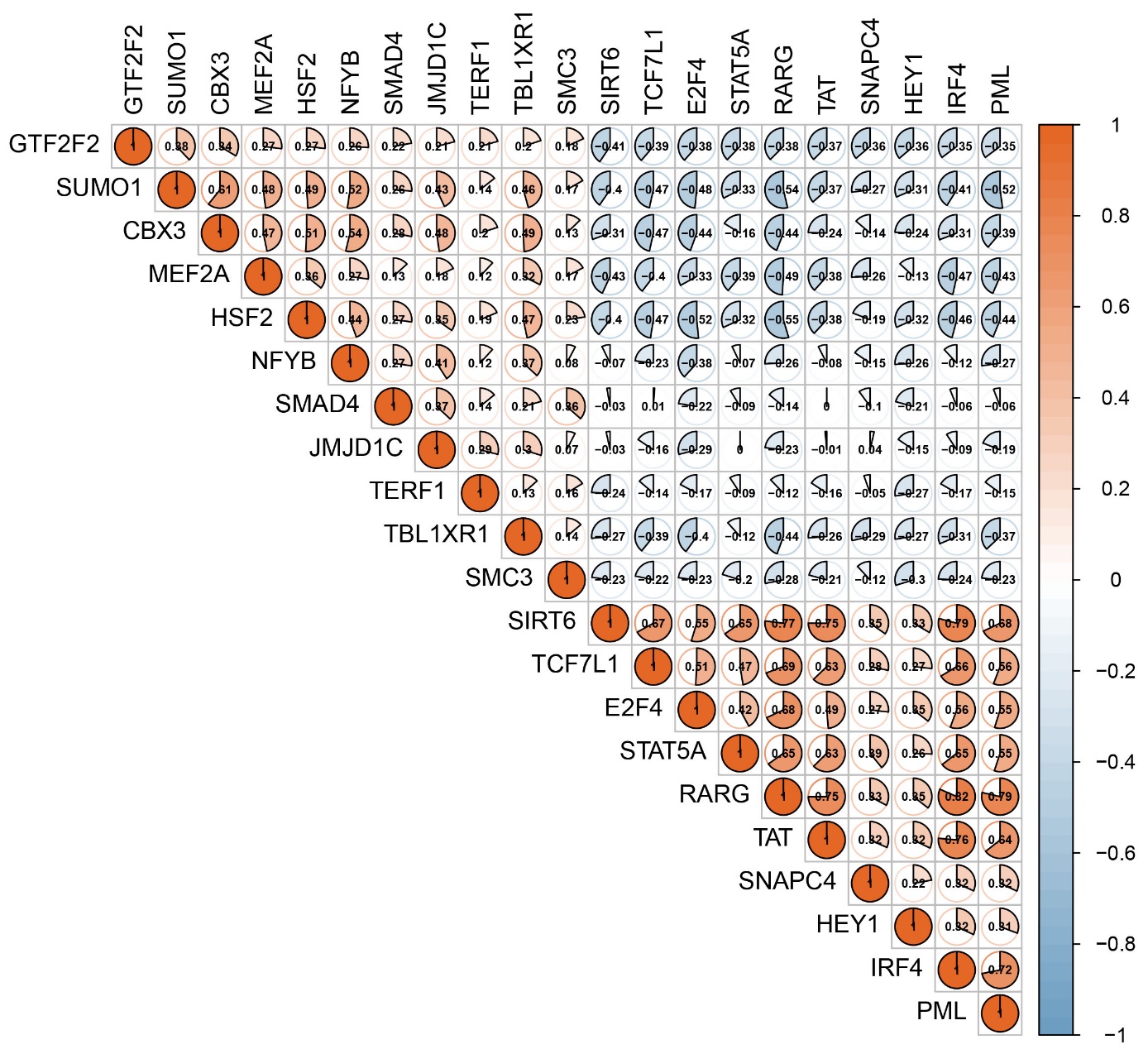


Figure S10. Correlation of GTF2F2 with TFs. Heatmap indicated GTF2F2 was associated with top10 up-regulated and top10 down-regulated TFs according to log2|fold change| between low and high GTF2F2 expression subgroups.


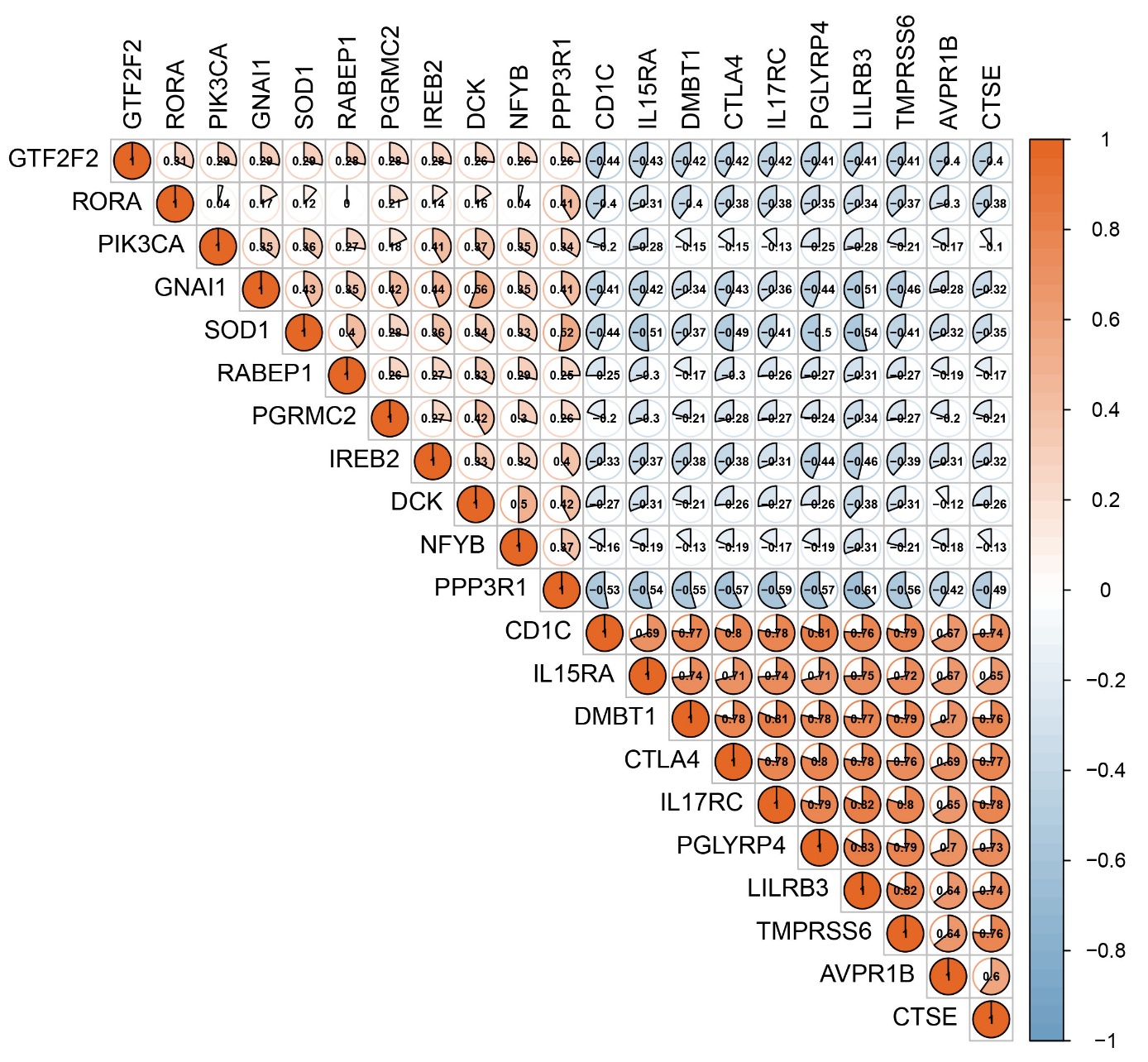


Figure S11. Correlation of GTF2F2 with immune genes. Heatmap indicated GTF2F2 was related to top10 up-regulated and top10 down-regulated immune genes based on log2|fold change| between low and high GTF2F2 expression subgroups.


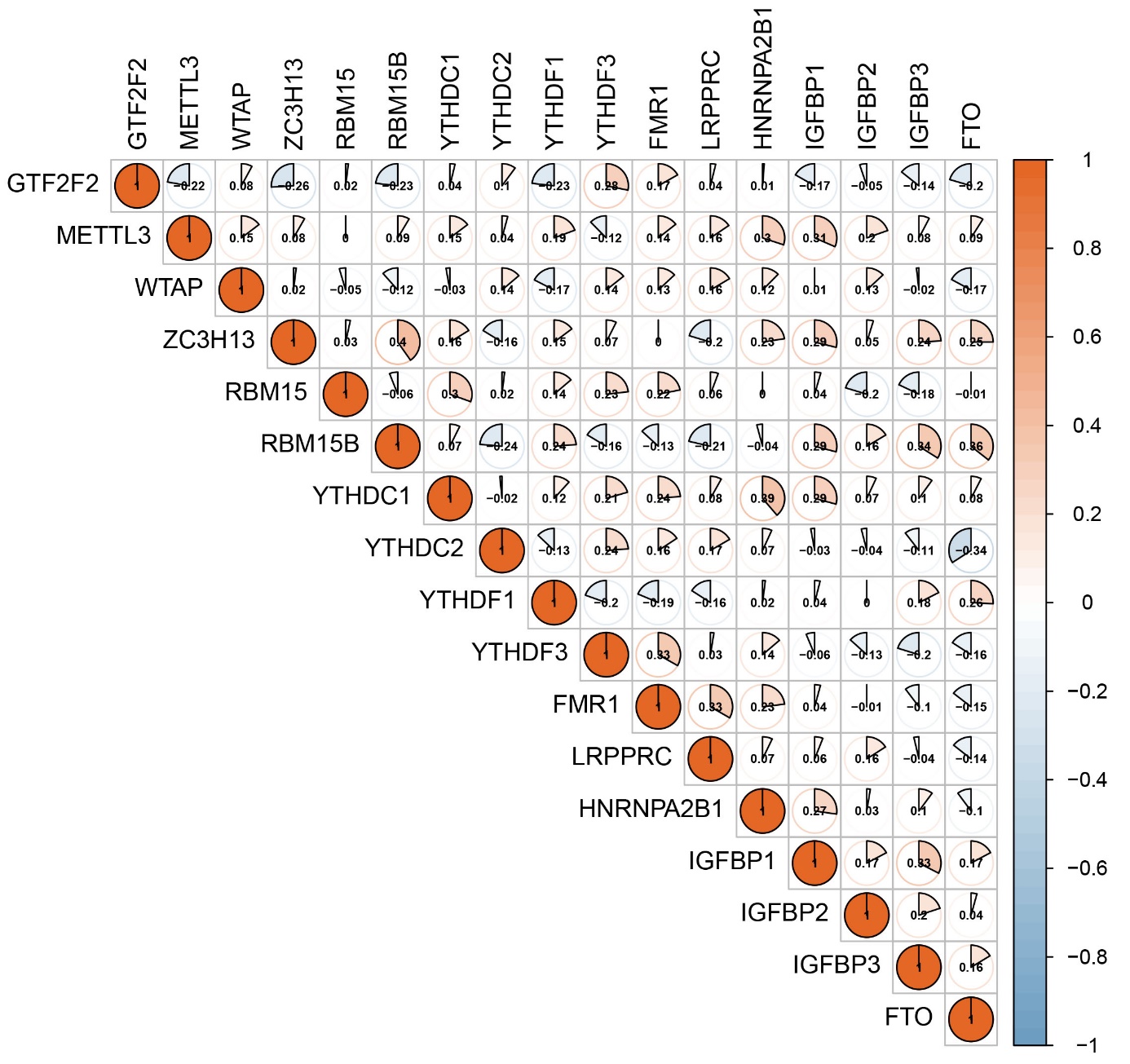


Figure S12. Association of GTF2F2 with m6A genes. Heatmap indicated GTF2F2 established correlation with top10 up-regulated and top10 down-regulated m6A genes according to log2|fold change| between low and high GTF2F2 expression subgroups.
